# Autobiographical Memory and End-of-Life Treatment Preferences in China

**DOI:** 10.1093/geroni/igaa057.2049

**Published:** 2020-12-16

**Authors:** Yao Fu, Ellen Idler

**Affiliations:** Emory University, Atlanta, Georgia, United States

## Abstract

In this mixed-methods study of religious/cultural beliefs and end-of-life treatment preferences in China, we surveyed 1,085 mainland Chinese people aged 18 or above online. We assessed the effects of past experience with dying people they have known and their own end-of-life treatment preferences in two hypothetical terminal illness vignettes. We found that respondents who knew or visited someone at the end of their lives were somewhat less likely to choose aggressive treatment for themselves in a lung cancer scenario (25% compared to 33%, p=.013). However, there was less difference in an Alzheimer’s disease scenario, with a choice to use a gastric feeding tube or not (39% compared to 42%, p=.262). Open-ended responses indicate that people refer to these past experiences as a reference in making end-of-life decisions for themselves. This study provides empirical evidence that autobiographical memory has a directive function that individuals call on to inform future behaviors.

